# Supplementary material for: Netcombin: An algorithm for constructing optimal phylogenetic network from rooted triplets
Source: PLoS One. 2020 Sep 18;15(9):e0227842. doi: 10.1371/journal.pone.0227842 (PMC7500971; doi:10.1371/journal.pone.0227842)
Supplement: S4 File — (ZIP) [file pone.0227842.s004.zip › SequenceData-Triplet/sequenceData-10-20-30-0-Triplet/length 500, numseq 30, rate 10^-7/4 seq.pdf]

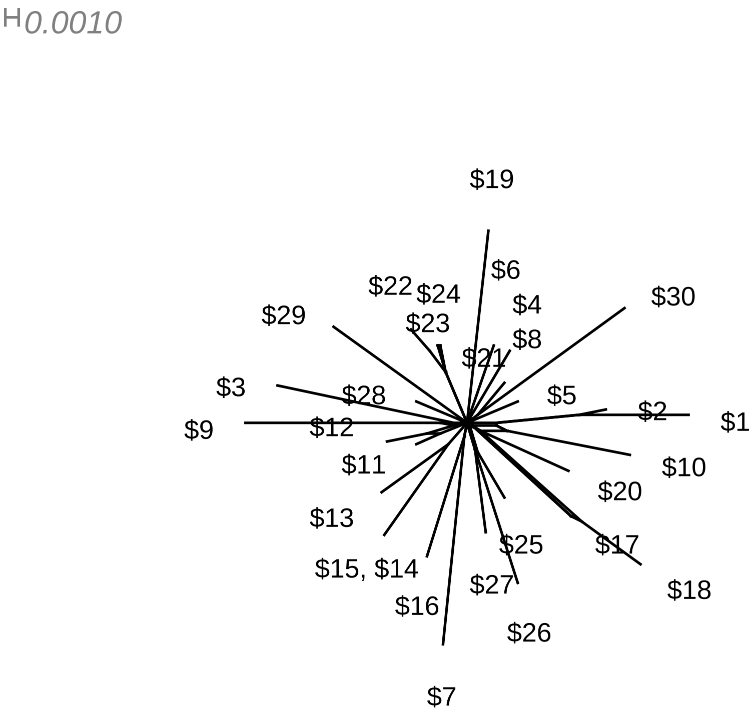
0.0010
$19
$6
$22
$24
$4
$30
$29
$23
$8
$21
$3
$28
$5
$2
$1
$9
$12
$11
$10
$20
$13
$25
$17
$15, $14
$27
$18
$16
$26
$7
